# CGIMP: Real-time exploration and covariate projection for self-organizing map datasets

**DOI:** 10.21105/joss.01520

**Published:** 2019-07-10

**Authors:** Adam G. Diehl, Alan P. Boyle

**Affiliations:** 1 Department of Computational Medicine and Bioinformatics, University of Michigan; 2 Department of Human Genetics, University of Michigan

## Summary

Dimensionality-reduction methods are widely used to break down complex datasets into more manageable subunits. For example, self-organizing maps (SOMs) (Kohonen, 1990), a type of neural network, are capable of projecting high-dimensional data onto a two-dimensional grid topography. Each grid cell (node) within these mappings represents a cluster of data points (modules) with similar properties, and the distance between nodes on the map is inversely correlated with their underlying similarity. These properties allow these maps to capture the properties of complex datasets and represent them in a human-readable form that facilitates further analysis. In particular, projecting covariates onto these mappings can yield insights into how and why modules cluster together, giving clues to their underlying properties and potential functions within the system from which they were drawn. For example, SOMs have been used in computational genomics to distill co-occurence data for large sets of DNA binding proteins into common co-binding patterns (Boyle et al., 2014; Diehl & Boyle, 2018; Xie et al., 2013). Projecting various genomic annotations onto these mappings has yielded insights into the biological processes and mechanisms associated with different co-binding patterns.

However, while multiple tools exist to produce SOMs and graphically render their results (Wehrens & Buydens, 2007; Yuan, 2018), none are designed for real-time data exploration and projection of covariate data, which generally requires additional steps outside the core software package. Furthermore, mapped outputs are static and non-interactive. Drilling down into the dataset generally requires manually obtaining slices of the data frame through a scripting language or API. Finally, making comparisons between maps is cumbersome, requiring preparation of multiple individual images through the same text-based interface. We have previously addressed this problem by producing a web application that harnessed the interactive capabilities of SVG map renderings to provide access to underlying data and facilitate map comparisons (Diehl & Boyle, 2018). However, this browser still required manual image preparation and was not designed with dataset portability in mind, limiting its utility.

(C)lustered (G)enomic (I)nterval (M)apping (P)latform (CGIMP) is a web application that addresses these limitations by enabling real-time analysis of self-organizing maps for genomics datasets. CGIMP takes two inputs: a JSON file describing the modules from a genomic dataset that has been classified and labeled by an SOM algorithm, and a separate JSON with descriptive data for each node in the map grid. Given these inputs, it will automatically render an interactive map image to the screen and provide a set of data-driven search facets that allow direct exploration of the intrinsic properties of the dataset. It also provides the ability to directly intersect the underlying data with covariate datasets uploaded to the server as BED files. These are intersected with the dataset through a python adapter to the popular BEDTools suite (Quinlan & Hall, 2010).

The CGIMP browser consists of four main components: the interactive map image, the data-driven faceted search area, the data upload component, and a display component providing interactive data tables for the selected map node. The map image and data tables are updated in real-time whenever the user makes changes to the search facets or triggers an intersection within the file upload area. Map images are interactive, with hover events revealing a tooltip with basic descriptive data for each node, while click events trigger updates to the data display component. Tooltips and data display tables can be customized through the settings panel, which also provides access to lower-level properties of the browser and dataset for advanced users. Furthermore, browser data for the current view, including map images, tabular data, and JSON data, can be saved for further analysis or publication with the click of a button.

CGIMP runs on a single Docker container, based on node.js and the React web framework, and is fully-portable. Its only external dependencies are a functioning Docker daemon and web server on the host machine. All intrinsic dependencies are either built in to the Docker image or installed using a configuration script included in the git repository. CGIMP ships with an example tab-delimited text dataset and a set of python scripts for transforming the dataset into the proper JSON form prior to loading. These scripts include internal comments to guide the necessary changes to tailor them to any arbitrary dataset. While CGIMP is designed for SOM datasets with a hexagonal grid topology, extending this tool to additional methods and topologies should, in principle, be possible with only minor modifications.

CGIMP is available on GitHub at https://github.com/Boyle-Lab/CGIMP, under the GNU GPL3 license. Detailed installation and usage instructions are available at https://cgimp.readthedocs.io/.
